# Impairment of the Retinal Endothelial Cell Barrier Induced by Long-Term Treatment with VEGF-A_165_ No Longer Depends on the Growth Factor’s Presence

**DOI:** 10.3390/biom12050734

**Published:** 2022-05-23

**Authors:** Heidrun L. Deissler, Matus Rehak, Armin Wolf

**Affiliations:** 1 Department of Ophthalmology, Ulm University Medical Center, 89075 Ulm, Germany; armin.wolf@uniklinik-ulm.de; 2 Department of Ophthalmology, Justus-Liebig-University Giessen, 35392 Giessen, Germany; matus.rehak@augen.med.uni-giessen.de

**Keywords:** retinal endothelial cells, VEGF-A, transcellular flow, paracellular flow, tight junction, cell adhesion, claudin-1, plasmalemma vesicle associated protein, tivozanib, nintedanib

## Abstract

As responses of immortalized endothelial cells of the bovine retina (iBREC) to VEGF-A_165_ depend on exposure time to the growth factor, we investigated changes evident after long-term treatment for nine days. The cell index of iBREC cultivated on gold electrodes—determined as a measure of permeability—was persistently reduced by exposure to the growth factor. Late after addition of VEGF-A_165_ protein levels of claudin-1 and CD49e were significantly lower, those of CD29 significantly higher, and the plasmalemma vesicle associated protein was no longer detected. Nuclear levels of β-catenin were only elevated on day two. Extracellular levels of VEGF-A—measured by ELISA—were very low. Similar to the binding of the growth factor by brolucizumab, inhibition of VEGFR2 by tyrosine kinase inhibitors tivozanib or nintedanib led to complete, although transient, recovery of the low cell index when added early, though was inefficient when added three or six days later. Additional inhibition of other receptor tyrosine kinases by nintedanib was similarly unsuccessful, but additional blocking of c-kit by tivozanib led to sustained recovery of the low cell index, an effect observed only when the inhibitor was added early. From these data, we conclude that several days after the addition of VEGF-A_165_ to iBREC, barrier dysfunction is mainly sustained by increased paracellular flow and impaired adhesion. Even more important, these changes are most likely no longer VEGF-A-controlled.

## 1. Introduction

Pathogenesis of various ocular diseases of high socio-economic relevance, e.g., macular edema secondary to diabetic retinopathy or retinal vein occlusion is associated with deregulated expression of growth factors and cytokines in the vitreous [1,2,3]. Of these, vascular endothelial growth factor (VEGF-A) plays a dominant role. Its splice variant VEGF-A_165_ not only induces angiogenic and inflammatory responses, but most importantly elevates the permeability of a monolayer formed by retinal endothelial cells (REC) in vitro [4,5,6,7,8]. Similar to the tight blood–brain barrier, the inner blood–retina barrier (BRB) is formed by REC, retinal pericytes and glia cells, and dysfunction of the inner BRB eventually results in macular edema [9,10,11].

Enhanced permeability of primary or immortalized REC induced by VEGF-A_165_ treatment for a few days is strongly associated with reduced expression of tight junction (TJ) protein claudin-1 and increased levels of the plasmalemma vesicle associated protein (PLVAP), indicative of deregulated paracellular and transcellular flow [6,7,8,12,13,14,15]. The presence of TJ-proteins claudin-5 and claudin-1 at the plasma membrane is patchy or lost, respectively, after treatment with VEGF-A_165_ for two days; during extended exposure to the growth factor for six days claudin-1 remains absent from the plasma membrane but claudin-5′s continuous distribution at the plasma membrane is re-established [15]. Delocalization of the adherens junction (AJ) protein vascular endothelial cadherin (VEcadherin) from the plasma membrane is mainly observed shortly after the addition of the growth factor to the endothelial cells (EC) [16,17,18]. Ragged VEcadherin-specific staining of the plasma membrane is also evident after exposure of REC to the growth factor for two days [15].

VEGF-A_165_-driven signal transduction is initiated by its binding to receptor tyrosine kinases VEGF receptors (VEGFR) 1/2 and the non-tyrosine kinase receptor Neuropilin-1 [19,20,21,22,23]. These receptors are expressed by REC, and activation of VEGFR2 by VEGF-A_165_ results in an increased permeability of REC monolayers [4,6,24]. Besides turning-on signal transduction mediated by mitogen-activated protein kinases and their targets, VEGF-A_165_ also activates the WNT/β-catenin-pathway: High permeability of VEGF-A_165_-treated human retinal microvascular endothelial cells (HRMEC) is associated with deregulated mRNA-expression of various members of the WNT-pathway including β-catenin which is more strongly expressed [25]. VEGF-A_165_ also induces translocalization of β-catenin to the nucleus in primary bovine retinal endothelial cells (BREC) [26].

Our previous findings suggest that VEGF-A_165_-induced barrier dysfunction of immortalized BREC (iBREC) is no longer dependent on the growth factor itself when cells have been exposed for up to five days. VEGF-A-binding proteins, i.e., the Fab fragment ranibizumab and the single chain variable fragment brolucizumab, completely prevent but only transiently revert VEGF-A_165_-induced dysfunction of the iBREC barrier, although their target is still completely bound by the antagonists even five days after their addition [15,27]. To better understand the differences of the early and late response of iBREC to VEGF-A_165_, we investigated changes of the expression levels of candidate proteins induced by treatment with the growth factor for up to nine days. These included proteins involved in regulation of para- and transcellular flow, i.e., claudin-1, claudin-5, VEcadherin, caveolin-1, and PLVAP, as well as in adhesion of iBREC to the extracellular matrix, i.e., tetraspanin CD9/TSPAN29, integrin α5/CD49e, and integrin β1 [6,7,8,12,13,14,15,24,28,29]. In addition, we assessed the capability of brolucizumab to prevent these late-phase changes and studied whether the time point of addition of small molecule inhibitors of VEGFR1/2, i.e., nintedanib and tivozanib, to VEGF-A_165_-exposed cells was important for their capacity to revert the detrimental changes induced by the growth factor [30,31,32]. In contrast to brolucizumab, which by binding all isoforms of VEGF-A prevents their interaction with the VEGF receptors, nintedanib and tivozanib inhibit their tyrosine kinase activity [31,32,33]. Both molecules specifically block VEGFR2 activity at a concentration of 10 nM (see Table 1) which is also sufficient to prevent VEGF-A_165_-induced dysfunction of the barrier formed by iBREC [18,24,31,32]. At higher concentrations (see Table 1), they additionally inactivate other receptor tyrosine kinases, i.e., VEGFR1, platelet derived growth factor receptors (PDGFRs) or fibroblast growth factor receptors (FGFRs), as well as cellular tyrosine kinases, all possibly activated by VEGF-A_165_ [31,32].

**Table 1:** Characteristics of inhibitors.

Inhibitor	Target Protein	IC50		Final Concentrations	Reference	Provider ^(1)^
**Nintedanib**	VEGFR1	34	nM	10 or 100 nM	[32]	Selleckchem
	VEGFR2	13	nM			
	VEGFR3	13	nM			
	FGFR1	69	nM			
	FGFR2	37	nM			
	FGFR3	108	nM			
	PDGFRα	59	nM			
	PDGFRβ	65	nM			
**Tivozanib**	VEGFR1	30	nM	10 or 100 nM	[31]	Selleckchem
	VEGFR2	6.5	nM			
	VEGFR3	15	nM			
	PDGFRα	40	nM			
	PDGFRβ	49	nM			
	c-Kit	48	nM			

^(1)^ Selleckchem: Selleckchem via Absource GmbH, Munich, Germany.

## 2. Materials and Methods

### 2.1. Cell Culture

Telomerase-immortalized microvascular endothelial cells from bovine retina (iBREC), established and characterized in our laboratory [27], were cultivated on fibronectin-coated surfaces (#356008, Corning, Amsterdam, The Netherlands or #F2006, Merck, Darmstadt, Germany) in Endothelial Cell Growth Medium MV (ECGM; Promocell, Heidelberg, Germany) containing 1 g/L glucose, 0.4% Endothelial Cell Growth Supplement/H, 90 µg/mL heparin, 10 ng/mL human epidermal growth factor (hEGF), 100 nM hydrocortisone, 5% fetal bovine serum (FBS; all supplements were from Promocell), and 300 µg/mL geneticin (Thermo Fisher Scientific, Langenselbold, Germany) as described in detail elsewhere [15,24,27,33]. Cells were used from passages 25 to 60 counting from the stage of primary culture. Their cultures contain >98% cells expressing proteins specific for (microvascular) EC, e.g., claudin-5, von Willebrand factor and are negative for α-smooth muscle actin-expressing cells [27,33]. We also routinely recorded their characteristic proliferation profile by measurements of the electric cell-substrate impedance with the microelectronic biosensor system for cell-based assays xCELLigence RTCA DP (Agilent, OLS, Bremen, Germany) [18].

All experiments were performed with confluent monolayers of iBREC formed after cultivation in ECGM for four days. Cell culture medium was then completely replaced by ECGM, lacking hEGF but containing 1 µg/mL fibronectin, before VEGF-A_165_ (recombinant human *Sf*21-expressed, #293VE, bio-techne, Wiesbaden, Germany; final concentration of 50 ng/mL) was added one day later. At indicated time points, inhibitors of VEGFR2 tivozanib or nintedanib (for details see Table 1; final concentrations: 10 or 100 nM) which were dissolved in dimethyl sulfoxide (Merck), or the VEGF-A-binding brolucizumab (Beovu; 120 mg/mL brolucizumab in 10 mM sodium citrate, 0.02% polysorbate-80, 5.8% sucrose, pH ~7.2; Novartis Pharma GmbH; Nuremberg, Germany; final concentration of brolucizumab: 1 mg/mL) were placed in the cell culture medium, i.e., VEGF-A_165_ was always present until the end of the experiment [15,24,30,31,32,33,34]. In control experiments, cells were processed in exactly the same way in cell culture medium only lacking the effector(s) investigated.

### 2.2. Cell Index Measurements

For assessing the stability of the barrier formed by a monolayer of iBREC cultivated on gold electrodes, we performed continuous electric cell-substrate impedance measurements with the microelectronic biosensor systems for cell-based assays xCELLigence RTCA DP as previously described [15,18,24,33,35]. Briefly, impedance was measured in each individual well of an E-Plate 16 PET (Agilent, OLS) between gold electrodes and expressed as the unit-free parameter cell index CI = (Z_i_ − Z_0_)/15 Ω (RTCA Software 2.0, Acea, OLS) with Z_i_ being the impedance measured at an individual time point and Z_0_ the impedance read at the start of the experiment [18,36]. Cells (~1 × 10^4^) were seeded per fibronectin-coated well and the cell index was measured every 15 min until a confluent cell monolayer was reached three to four days later, indicated by a constantly high cell index (CI~18). Then, the cell culture medium was completely exchanged with ECGM lacking hEGF but containing 1 µg/mL fibronectin, and the cell index was assessed every 15 min. VEGF-A_165_ was added at a final concentration of 50 ng/mL one day later, and tivozanib, nintedanib, or brolucizumab at indicated time points. The cell index was measured every five minutes until the end of the experiment nine days after addition of VEGF-A_165_. Recorded CI values (*n* ≥ 4 for each condition and time point) were normalized in relation to those measured immediately before addition of the VEGF-A_165_ (RTCA Software Pro 2.3.4 (Basic), Agilent, OLS, Santa Clara, CA, USA), and the results were converted to graphs showing means and standard deviations with Graph Pad Prism 6 (Graph Pad Software, San Diego, CA, USA) [15,18,24,33,35]. Experiments were independently performed three times.

### 2.3. Preparation of Cell Extracts and Subcellular Fractions and Western Blot Analyses

Confluent monolayers of iBREC were exposed to VEGF-A_165_ before cells and cell culture supernatants were harvested at indicated time points. Alternatively, cells were pre-treated with VEGF-A_165_ for one day and subsequently exposed to brolucizumab for eight days. Whole cell extracts or subcellular fractions yielding proteins associated with the cytoplasm, the membranes and organelles or the cytoskeleton were prepared as described previously in detail [33,35]. The NE-PER Nuclear and Cytoplasmatic Extraction Reagents (#78833, Thermo Fisher Scientific, Waltham, MA, USA) were used for preparation of nuclear extracts according to the manufacturer’s instructions. Cells (2 × 10^6^) were suspended in 150 µL CER I supplemented with EDTA-free HALT Protease Inhibitor Cocktail (#78437, Thermo Fisher Scientific) by vigorously mixing for 15 s. After incubation on ice for 10 min, 8.25 µL CER II were added, the suspension vortexed for five seconds, and placed on ice for one minute followed by vigorously mixing for five seconds. The supernatant (→ cell extract) of the subsequent centrifugation (5 min, 16,000× *g*, 4 °C) was carefully removed and the cell pellet resuspended in 75 µL NER supplemented with EDTA-free HALT Protease Inhibitor Cocktail by vigorously mixing for 15 s. Samples were placed on ice for 10 min and mixed again vigorously for 15 s; this step was repeated three times. After centrifugation for 10 min at 16,000× *g* and 4 °C, the supernatant (→ nuclear extract) was carefully removed. All protein extracts were prepared at least three times for each condition and were stored at −80 °C. Protein concentrations were determined using the BCA Protein Assay Kit (Thermo Fisher Scientific, #23227).

We analyzed proteins of potential relevance by Western blotting of whole cell extracts, but subcellular fractions containing proteins from membranes and organelles or nuclear extracts were used to measure PLVAP or β-catenin, respectively [15,18,33]. Proteins were always separated under reducing conditions, only for analyzing CD9/TSPN29, CD29/integrin β1, or VEGF-A were non-reducing conditions applied; primary and secondary antibodies are listed in Table 2.

Chemiluminescence signals were directly scanned with the imaging system Fusion Pulse TS (Vilbert Lourmat, VWR, Darmstadt, Germany) and in case of whole cell extracts, peak volumes were set in relation to those obtained for actin in the very same sample or in case of protein fractions to the protein concentration. To compare results of multiple Western blot experiments, signals of effector-treated cells were normalized to those obtained from similarly processed control cells (=1) with the exception of VEGF-A which were normalized to those from VEGF-A_165_-exposed cells (=1) [18,33,35].

### 2.4. Determination of VEGF-A

Concentrations of VEGF-A were determined in cell culture supernatants with the Quantikine ELISA Canine VEGF-A Immunoassay Kit (CAVE00, bio-techne), which can be used to quantify human, canine, and bovine VEGF-A, as previously described [15,18,33,35]. Samples were diluted 1:100 with PBSd (cell culture supernatants of VEGF-A-exposed cells) or processed undiluted (all other cell culture supernatants) [15,18]. Triplicate or quadruplicate samples were processed according to the manufacturers’ instructions and analyte-dependent absorbance was measured at 450 nm (reference wavelength: 570 nm) 10–20 min after addition of the stop solution with an Infinite 200Pro spectrophotometer controlled by Tecan i-control software (Tecan, Crailsheim, Germany) [33,35]. Standard curves (0 to 1250 pg/mL VEGF-A_165_ included in the ELISA kit) were generated in parallel to the analyses of samples to allow accurate quantifications of more than 20 pg/mL VEGF-A. Assays were performed three times with independently prepared samples.

### 2.5. Immunofluorescence Stainings

Cells were cultivated on fibronectin-coated two-chamber slides (x-well PCA Tissue Culture Chambers; Sarstedt, Nuembrecht, Germany) and exposed to VEGF-A_165_ before slides were fixated at indicated time points in methanol for 7.5 min at −20 °C. After blocking, presence of β-catenin was determined with specific antibodies (see Table 2) followed by incubation with Alexa Fluor595-labeled secondary antibodies (goat F(ab′)_2_ fragment directed against rabbit IgG, heavy and light chains, Thermo Fisher Scientific, #A11072; diluted 1:500) as described in detail elsewhere [29,33]. Afterwards, slides were embedded in ProLong Gold Antifade Mountant (P36935; Thermo Fisher Scientific) with 4′,6-diamidino-2-phenylindole (DAPI; λ_ex_/λ_em_ = 359 nm/461 nm) for examination with a fluorescence microscope (model DM4000B, software LAS X, Leica, Wetzlar, Germany) [15,29].

### 2.6. Statistical Analyses

The Mann–Whitney U test was applied when antigen-specific Western blot signals from differently treated cells were compared. For comparing signals from effector-treated cells and the hypothetical value of 1.00 of normalized signals from control cells, we used the One-sample t-test. In this type of statistical analysis, the variability of the values obtained from control cell signals is taken into consideration, although they appear without standard deviations (SD = 0). One-way analyses of variance (ANOVA) followed by Tukey’s test were used to compare groups of antigen-specific absorbances from ELISA. Data obtained by cell index measurements were analyzed with two-way ANOVA followed by Sidak’s multiple comparison test. Differences resulting in *p*-values below 0.05 were considered significant. All statistical analyses were performed with Graph Pad Prism 6; means and standard deviations are provided as numbers, graphs, or in scatter plots.

## 3. Results

### 3.1. VEGF-A_165_-Induced Persistent Impairment of the iBREC Barrier Was Associated with Changes of the Expression Levels of TJ-Protein Claudin-1 and Intgrins α5 and β1

**Figure 1:**
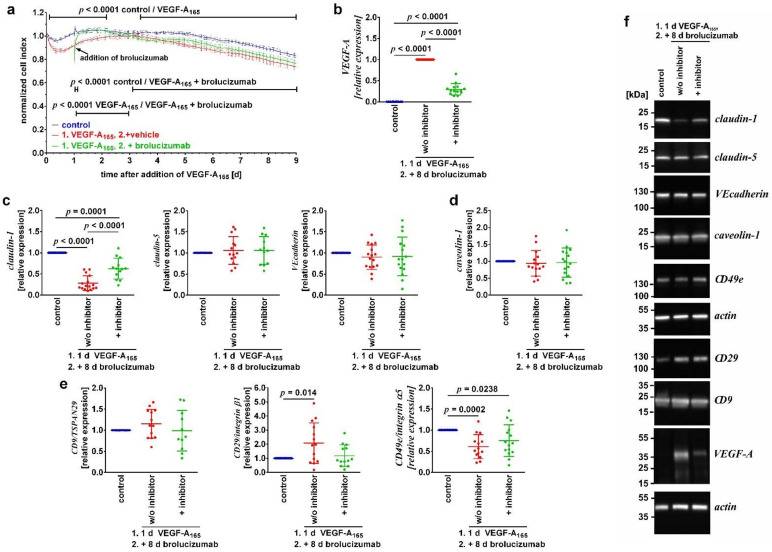
Prolonged treatment with VEGF-A_165_ impaired the barrier formed by iBREC. Confluent monolayers of iBREC were exposed to VEGF-A_165_ for nine days and (**a**) the cell index was measured continuously or (**b**–**f**) cells were harvested. Alternatively, iBREC were treated with the growth factor for one day, before the VEGF-A antagonist brolucizumab was added for an additional eight days. (**a**) VEGF-A_165_ induced a persistent decrease in the cell index, which was quickly reverted by brolucizumab, but this effect was only stable for 1.5 days. Cell index values obtained from more than five wells per condition were normalized to those measured just before the addition of VEGF-A_165_ and are shown as means ± standard deviations. Data were analyzed as described in “Materials and Methods”. (**b**) Less VEGF-A was taken up by iBREC in the presence of brolucizumab. (**c**) Levels of TJ-protein claudin-1 were low after exposure of the cells to the growth factor in the absence or presence of its antagonist. (**d**) None of the effectors changed expression levels of caveolin-1. (**e**) VEGF-A_165_ induced higher levels of CD29/integrin α1 but lower levels of CD49e/integrin α5 not changed by brolucizumab. Signals obtained by Western blot analyses were normalized and statistical analyses performed as described in “Materials and Methods”. Pooled results of multiple Western blot experiments are shown as scatter plots also depicting means ± standard deviations where one dot represents the single signal obtained from one experiment. (**f**) Representative images of Western blot analyses; the original images are shown in Figure S1.

In order to study the changes of the barrier formed by a monolayer of iBREC caused by prolonged treatment with VEGF-A_165_, cells were incubated with the growth factor for up to nine days. Continuous determination of the cell index revealed that the growth factor induced a significant decrease in the cell index lasting until the end of the experiment, nine days after its addition (Figure 1a, compare blue and red curves).

To determine whether the amount of exogenously added VEGF-A changed during long-term exposure, we measured its extracellular concentration by competitive ELISA. The detection antibody competes with proteins binding to VEGF-A, e.g., VEGFR1 and VEGFR2, therefore only unbound growth factor is measured [37,38]. Free VEGF-A was only present in supernatants of VEGF-A-exposed cells and, nine days after its addition, its levels were about 10% of the amount exogenously added (6 ± 0.9 ng/mL for VEGF-A_165_-exposed cells; N = 12).

As instability of the barrier formed by iBREC is associated with increased paracellular and transcellular flow, as well as impaired adhesion, we assessed expression levels of candidate proteins in cells exposed to VEGF-A_165_ for nine days by Western blot analyses. These included proteins regulating paracellular (i.e., claudin-1, claudin-5, and VEcadherin) or transcellular (i.e., caveolin-1 and PLVAP) flow, and cell adhesion proteins (i.e., tetraspanin CD9/TSPAN29 and subunits of the fibronectin receptor CD29/integrin β1 and CD49e/integrin α5) (Figure 1c–f, compare blue and red signals). Expression levels of claudin-1 (Figure 1c) were significantly lower compared to control whereas those of claudin-5, VEcadherin, and caveolin-1 (Figure 1d) were not different from those of control cells. After exposure of iBREC to VEGF-A_165_ for up to six days, levels of PLVAP isolated together with proteins from membranes and organelles are significantly higher, whereas the protein is not detected in subcellular fractions of unchallenged cells [15]. Interestingly, PLVAP was no longer present in the subcellular fractions of proteins from the cytoplasm, the membranes and organelles, or the cytoskeleton, after extended treatment of iBREC with the growth factor for nine days. Expression levels of proteins important for adhesion of iBREC to the extracellular matrix were altered in different ways by long-term treatment (Figure 1e); the tetraspanin CD9/TSPAN29 was stably expressed, but levels of the subunits of the fibronectin receptor CD29/integrin β1 and CD49e/integrin α5 were changed in opposite ways. The former was significantly stronger expressed, the latter significantly weaker.

**Figure 2:**
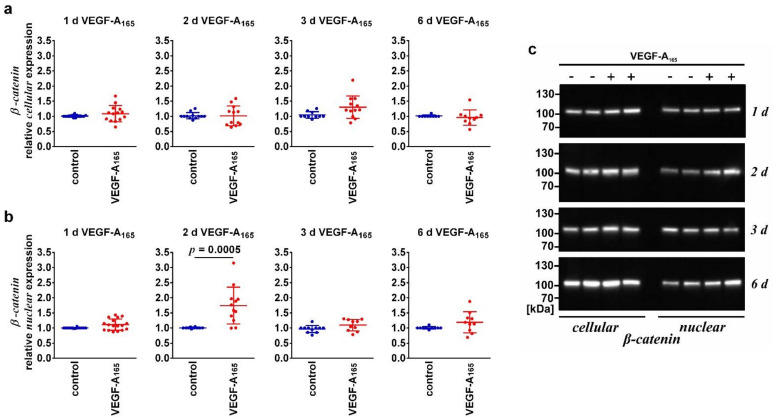
Nuclear expression levels of β-catenin changed over time of exposure to VEGF-A_165_. Confluent iBREC were exposed to VEGF-A_165_ and cells were harvested one to six days later for preparation of (**a**) cellular and (**b**) nuclear extracts and subsequent Western blot analyses of which representative images are shown in (**c**). Levels of (**a**) cellular β-catenin remained unchanged by VEGF-A_165_ but (**b**) those of nuclear β-catenin were significantly elevated only on day 2. Signals obtained by Western blot analyses were normalized and analyzed as described above. Please refer to Figure S2 for original images.

To evaluate whether the WNT/β-catenin-pathway was indeed activated by VEGF-A_165_, we studied expression levels of β-catenin and its subcellular localization in iBREC exposed to the growth factor for one to six days. Cellular levels of β-catenin were not changed (Figure 2a) and its nuclear expression levels were elevated only on day 2 after the addition of the growth factor (Figure 2b).

**Figure 3:**
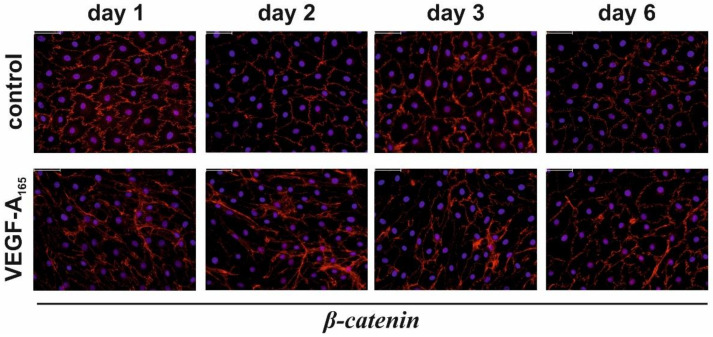
Plasma membrane localization of β-catenin was impaired by extended exposure of iBREC to VEGF-A_165_. Confluent cells were exposed to VEGF-A_165_ and cells were fixated at indicated time points. Subcellular localization of β-catenin was visualized by immunofluorescence stainings with specific antibodies (red); nuclei were stained with DAPI (blue). In unchallenged cells, most of β-catenin was present at the plasma membrane, but exposure to VEGF-A_165_ for one to three days resulted in its de-localization from the plasma membrane, less evident after extended exposure for six days. Scale bar 50 µm.

Immunofluorescence staining revealed that the vast amount of β-catenin was localized at the plasma membrane of unchallenged iBREC (Figure 3). This specific staining was disrupted after exposure of the cells to VEGF-A_165_.

### 3.2. Binding of VEGF-A Was Not Sufficient to Sustainably Revert Its Detrimental Long-Term Changes

Confluent iBREC were exposed to VEGF-A_165_ for one day before VEGF-A-binding brolucizumab was added for another eight days. The low cell index induced by treatment with the growth factor increased within a few hours, but this effect was only stable for a few days; eight days after addition of the VEGF-A antagonist, curves of cells treated with VEGF-A_165_ only and those of cells subsequently exposed to brolucizumab were nearly identical (Figure 1a, compare red and green curves).

To evaluate whether unbound VEGF-A—possibly available for signal transduction—was present in the supernatants of iBREC exposed to the growth factor plus brolucizumab, the amount of non-complexed VEGF-A was assessed by competitive ELISA; however, it was below the detection limit. Denaturing Western blot analyses were applied to determine the amount of total VEGF-A in supernatants or cell extracts of effector-treated iBREC, because proteins possibly interacting with the growth factor cannot interfere with its detection under these conditions. Extracellular VEGF-A was barely detectable, but its levels were significantly higher when cells had been exposed to VEGF-A_165_ and subsequently to brolucizumab (normalized values were: 1 ± 0 for VEGF-A_165_ and 3.01 ± 1.90 for VEGF-A_165_ + brolucizumab; *p* = 0.02; N = 8 for each condition). Less VEGF-A was also internalized by the cells when they had been treated with VEGF-A_165_ and subsequently with its antagonist compared to cells exposed to the growth factor only (Figure 1b).

In accordance with the low cell index observed eight days after addition of brolucizumab to VEGF-A_165_-exposed cells, expression levels of claudin-1 (Figure 1c) and CD49e/integrin α5 (Figure 1e) were still low; those of other proteins investigated remained unchanged.

### 3.3. Inhibitors of VEGFR2 Only Reverted VEGF-A_165_-Induced Dysfunction When Added during the Early Phase of the Growth Factor’s Action

**Figure 4:**
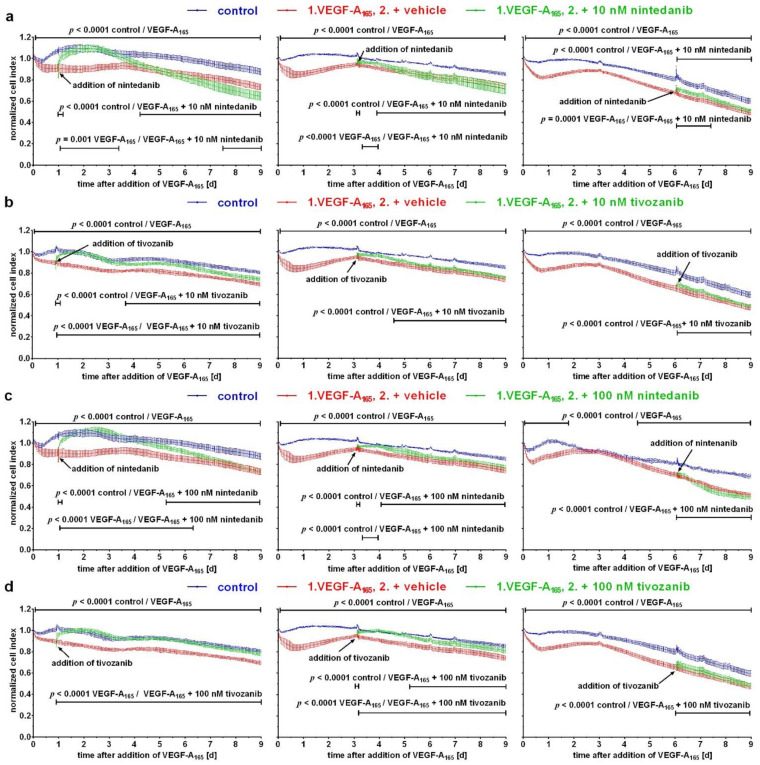
Inhibition of only VEGFR2 was not sufficient to normalize late VEGF-A_165_-induced barrier dysfunction. After exposure of confluent monolayers of iBREC cultivated on gold electrodes to VEGF-A_165_, (**a**,**b**) 10 nM or (**c**,**d**) 100 nM, nintedanib or tivozanib were added at indicated time points, and the cell index was measured continuously. Cell index values obtained from more than five wells per condition were normalized to those measured just before the addition of VEGF-A_165_ and are shown as means ± standard deviations. (**a**,**b**) Only when added during the early phase of VEGF-A-exposure were 10 nM nintedanib or tivozanib able to revert the induced changes, although these effects were not long-lasting. (**c**,**d**) When added during the early phase of VEGF-A-exposure, tivozanib, but not nintedanib, completely and sustainably normalized induced changes. Both inhibitors had little or no effect when added later.

To confirm our hypothesis that the late response of iBREC to exposure to VEGF-A_165_ is independent of the presence of the growth factor itself, we investigated whether the efficiency of inhibitors of the VEGFR2 tivozanib and nintedanib depends on the time point of their addition. Inhibitors were used at a concentration of 10 nM, specifically inhibiting VEGFR2 (see Table 1), which is also sufficient to revert the VEGF-A_165_-induced low cell index [24,29,31,32]. In accordance with our previous observations, 10 nM nintedanib (Figure 4a) and 10 nM tivozanib (Figure 4b) normalized the low cell index values within a few hours when both were added to iBREC exposed to VEGF-A_165_ for one day [24,29]. However, this effect was only stable for about two days. When iBREC had been pre-treated with VEGF-A_165_ for three or six days, the addition of both inhibitors did not result in a marked, if any, increase in the low cell index values (Figure 4a,b).

At higher concentrations, both inhibitors additionally block the activity of the receptor tyrosine kinasesVEGFR1 and PDGFRα/β [31,32]. Nintedanib also inhibits FGFR1/2/3, whereas the tyrosine kinase c-kit is inactivated by tivozanib (see also Table 1) [31,32]. Low cell index values were completely normalized within hours after addition of 100 nM nintedanib when added to cells exposed to VEGF-A_165_ for one day (Figure 4c). This effect was not long-lasting, and the addition of 100 nM nintedanib to cells treated with VEGF-A_165_ for three or six days was even less efficient, or did not have any effect at all, respectively (Figure 4c). Interestingly, 100 nM tivozanib completely and sustainably normalized low cell index values when added to iBREC exposed to the growth factor for one day (Figure 4d). When added later, 100 nM tivozanib—similar to nintedanib—only partly and transiently increased low cell index values or did not have any effect at all.

Taken together, in the late phase of the growth factor’s action, the inhibition of VEGFR2 was no longer sufficient to revert VEGF-A_165_ detrimental changes, and additional blocking of other receptor tyrosine kinases was at best only partially effective.

## 4. Discussion

VEGF-A-induced dysfunction of the barrier formed by REC has been studied in great detail, but research has been focused on the cells’ responses observed within minutes or hours after addition of the growth factor [5,16,17,19,21]. Our recently published data, however, suggest that changes evident after a few days differ markedly from these early responses [15]. Therefore, we investigated the long-term changes induced by VEGF-A_165_ between two and nine days after its addition in more detail. In order to prevent non-physiological responses of the cells due to serum deprivation and lack of nutrients, a cell culture medium adapted to the special needs of microvascular EC supplemented with FBS was used in all long-term experiments [33,35,39]. Cell index values were still high (CI~15) at the end of the experiments, confirming that observed changes are not caused by possibly cytotoxic metabolites; cells’ responses would then be expressed as very low values (CI~0.1) [40]. According to our preliminary findings, iBREC also did not secrete relevant amounts of IL-6, a marker of cellular stress, at any time point investigated, confirming that changes observed are solely due to the effectors added to the cells [41]. Responses of iBREC to VEGF-A_165_ are summarized in Table 3 and Table 4, also showing data of our previously published work [15,35].

**Table 3:** Responses of iBREC to VEGF-A_165_ depend on exposure time: concentrations of VEGF-A_165_ in cell culture supernatant.

Target	Incubation with VEGF-A_165_ for			
	1 Day	2 Days	6 Days	9 Days
VEGF-A_165_	36 ± 1.8 ng/mL	25 ± 3.4 ng/mL [15]	14 ± 1.2 ng/mL [15]	6 ± 0.9 ng/mL

**Table 4:** Responses of iBREC to VEGF-A_165_ depend on exposure time: changes of protein expression levels compared to cells not exposed to the growth factor.

Target	Incubation with VEGF-A_165_ for ^(1)^			
	1 Day	2 Days	6 Days	9 Days
Regulation of paracellular and transcellular flow:				
Claudin-1	↓↓ [35]	↓↓ [15,35]	↓↓ [15]	↓↓
Claudin-5	Unchanged [35]	Unchanged [15,35]	↑ [15]	Unchanged
VEcadherin	Unchanged [35]	Unchanged [15,35]	Unchanged [15]	Unchanged
Caveolin-1	Unchanged [35]	Unchanged [15,35]	Unchanged [15]	Unchanged
PLVAP	Not detected	↑↑ [15]	↑↑↑ [15]	Not detected
Adhesion to extracellular matrix:				
CD9/TSPAN29	Unchanged	Unchanged [15]	Unchanged [15]	Unchanged
CD29/integrin β1	↑	↑ [15]	↑ [15]	↑
CD49e/integrin α5	↑	↑ [15]	Unchanged [15]	↓
WNT-pathway:				
β-Catenin (nuclear)	Unchanged	↑	Unchanged	
β-Catenin (cellular)	Unchanged	Unchanged	Unchanged	

^(1)^ Expression levels were lower (↓), strongly reduced (↓↓), higher (↑), strongly (↑↑) or dramatically (↑↑↑) increased.

The significant decline of the cell index—assessed as a measure of barrier stability—induced by VEGF-A_165_ lasted for at least nine days and was accompanied by a steady decline of the exogenously added growth factor in the cell culture supernatant (Table 3). Uptake and degradation of the growth factor has been observed in EC including iBREC [20,35]. It is of interest that several days after the addition of VEGF-A_165_, the concentration of the unbound, extracellular growth factor was below the threshold of 25 ng/mL (~0.6 nM) needed to induce a dysfunction of the iBREC barrier [6]. Lower amounts of the growth factor might be sufficient to maintain the barrier dysfunction, and the permeability-inducing capacity of the remaining VEGF-A_165_ could be amplified by cytokines IL-6, IL-8, or TNFα [42,43,44]. However, this mechanism is unlikely because our preliminary data show that they were not secreted by iBREC during exposure to the growth factor.

Free, unbound VEGF-A was not detected in supernatants of cells exposed to the growth factor and its antagonist by competitive ELISA, indicating that the complex of brolucizumab and VEGF-A_165_ was stable over several days. However, the VEGF-A antagonist was not able to prevent the late barrier dysfunction, including VEGF-A_165_-induced changes of expression levels of claudin-1 or integrin α5/CD49e, when added in the early phase of VEGF-A_165_ action (Figure 1). Together with our finding that specifically blocking the tyrosine kinase activity of VEGFR2 was also not sufficient to normalize the late VEGF-A_165_-induced dysfunction of the barrier, these observations support our hypothesis that VEGF-A-driven signal transduction is no longer involved in the late phase of VEGF-A exposure. Additional inhibition of VEGFR1 by 100 nM nintedanib only transiently reverted VEGF-A-induced barrier dysfunction, and this is in accordance with our previous findings that its ligands placenta growth factors (P*l*GF) 1/2 did not impair the barrier formed by iBREC; binding of P*l*GFs by aflibercept would likely be no more efficient [7,45,46]. Possible activation of other signal transduction pathways driven by growth factors PDGFs or FGFs during extended exposure to VEGF-A_165_ is also rather unlikely because additional inhibition of receptor tyrosine kinases FGFR1/2/3 or PDGFRα/β by 100 nM nintedanib was also not more efficient. Basic FGF, PDGF-AA, or PDGF-BB by themselves do not destabilize the barrier formed by iBREC [6], unpublished observations. In contrast, 100 nM tivozanib maintained the normalized high cell index values when added to iBREC exposed to VEGF-A_165_ for one day; this might simply be due to an increased intracellular stability of the small molecule inhibitor. On the other hand, tivozanib also blocks tyrosine kinase c-kit, and its activation by stem cell factor (SCF) in turn results in an elevated permeability of an HRMEC monolayer completely prevented by inhibiting the tyrosine kinase’s activity [31,47]. Dysfunction of the barrier formed by HRMEC was evident within minutes after exposure to SCF and lasted for at least two hours; long-term changes were, however, not studied [47]. Nevertheless, possible activation of c-kit seems to be mainly relevant during the early phase of growth factor’s action, because tivozanib was not able to completely and sustainably revert late VEGF-A_165_-induced changes.

Activation of the WNT/β-catenin signaling pathway by VEGF-A_165_ has been shown, and indeed, we observed that more β-catenin was present in the protein fraction of nuclear proteins, but only two days after addition of VEGF-A_165_ (Table 4) [25,26]. In accordance with observations made by others, the very major amount of β-catenin was still localized at the plasma membrane, even during exposure to VEGF-A_165_ [26]. Under these conditions, β-catenin is transcriptionally inactive. Therefore, β-catenin-driven regulation of transcription is, most likely, irrelevant during the late phase of VEGF-A exposure. However, β-catenin binds directly to the AJ-protein VEcadherin, and VEGF-A_165_ similarly disrupts their localization at the plasma membrane, eventually resulting in dysfunction of the cells’ barrier [15,48].

Expression levels of proteins involved in the regulation of paracellular and transcellular flow varied during prolonged exposure of iBREC to VEGF-A_165_. Whereas TJ-protein claudin-1 was expressed at very low levels (~0.33% of control cells) from day 1 to day 9, expression of claudin-5 peaked at day 6 (see Table 4), but was similar to those of control cells at all other time points [15]. That VEGF-A_165_ enhances expression of claudin-5 over time has also been shown for primary BREC, although the presence of claudin-5 in the plasma membrane was then weaker compared to control cells [26]. Increased expression of PLVAP correlates with deregulated transcellular flow, resulting in barrier destabilization, and indeed the protein is expressed at high levels during prolonged treatment with VEGF-A_165_, but—surprisingly—is no longer detected very late after addition of the growth factor [12,13,14,15]. Therefore, changes of the barrier formed by REC evident late after addition of VEGF-A_165_ seem to be mainly due to deregulated paracellular flow resulting from an altered composition of TJ-complexes. Of these, claudin-1, which in contrast to claudin-5 is not specifically expressed in microvascular EC, plays a major role, because its reduced expression levels clearly correlate with a low cell index and therefore a dysfunctional barrier; transient overexpression of claudin-5 is not sufficient to counteract the loss of barrier function [15,49,50]. VEGF-A_165_ also destabilizes adhesion of iBREC to the extracellular matrix late after its addition; due to opposing changes of expression levels of CD29/integrin β1 and CD49e/integrin α5 possibly resulting in an unbalanced stoichiometry, formation of the fibronectin receptor by the two subunits might be hindered.

Taken together, we provide strong evidence that dysfunction of the barrier formed by REC caused by prolonged treatment with VEGF-A_165_ over several days is no longer dependent on the presence of the growth factor itself or signal transduction driven by VEGF-A. Blocking the growth factor’s interaction with its receptors or inhibiting their tyrosine kinase activities—even in addition to other growth factor receptors—is no longer sufficient to normalize the barrier function; a relevant contribution of growth factors P*l*GF1/2, PDGF, or FGFs can therefore be excluded [6,7,24]. As AJ and TJ provide the basis of a stable barrier formed by REC, a better understanding of the establishment of these junctions is mandatory and will likely help to identify relevant targets for counteracting the detrimental changes induced by VEGF-A [7,8,9,10,15,16,17,18]. Of these, the TJ-protein claudin-1 seems to be a promising candidate due to its persistent down-regulation during long-term exposure to VEGF-A_165_.

Although bovine cells were used in this study, previous research has shown that REC—either primary or immortalized—originating from different species, including human, similarly respond to growth factor stimulation [5,6,7,8,12,13,14,15,16,17,18,26]. Interactions with retinal pericytes and/or Müller cells likely affect the properties of the barrier expressed by REC, and indeed BREC cultivated in the presence of bovine retinal pericytes and rodent astrocytes expressed a slightly stronger barrier compared to that of the BREC monoculture [10,11,51]. Assessing VEGF-A_165_ detrimental effects on REC in the presence of the cells comprising the neurovascular unit might provide results more relevant to the in vivo situation and should be part of further studies.

VEGF-A-induced impairment of the inner BRB is a hallmark of macular edema secondary to diabetic retinopathy, but up to 30% of these patients do not adequately respond to anti-VEGF therapy [11,52,53]. If antagonists of VEGF were applied at a time point when dysfunction of the inner BRB was no longer solely dependent on VEGF-A_165_, an inadequate response could possibly result; increasing the concentration of VEGF-binding proteins would also be pointless under these conditions. Therefore, further studies are required to elucidate a better understanding of the mechanism behind VEGF-A’s prolonged action and to improve the outcomes of the treatment of diabetic macular edema.

## Figures and Tables

**Table 2:** Primary and secondary antibodies.

Target	Host, Type or Conjugate	Source ^(1)^	Working Concentrations
Actin	mouse, monoclonal	clone 5J11: Abcam, #ab190301 or bio-techne, #NBP2-25142	700 ng/mL
β-Catenin	rabbit, monoclonal	clone 6B3: Cell Signaling Technology B.V., #9582S	1:100
Caveolin-1	rabbit, polyclonal	Abcam, #ab2910	20 ng/mL
CD9	mouse, monoclonal	clone IVA: Exbio	40 ng/mL
CD29	mouse, monoclonal	clone TS2/16: Thermo Fisher Scientific, #14-0299-82	170 ng/mL
Claudin-1	rabbit, polyclonal	Thermo Fisher Scientific, #51-9000	0.25 µg/ml
Claudin-5	rabbit, polyclonal	Thermo Fisher Scientific, #34-1600	100 ng/mL
PLVAP	rabbit, polyclonal	Thermo Fisher Scientific, #PA5-110183	6 µg/ml
VEcadherin	rabbit, polyclonal	Cell Signaling Technology B.V., #2158S	1:1000
VEGF-A	goat, polyclonal	bio-techne, #AF1603	1:2000
Whole IgG, rabbit	goat, polyclonal, coupled to HRP ^(2)^	Biorad, #170-5046	1:30,000
Whole IgG, mouse	goat, polyclonal, coupled to HRP ^(2)^	Biorad, #170-5047	1:30,000
IgG, H + L chains, goat	donkey, polyclonal, coupled to HRP ^(2)^	bio-techne, #HAF109	1:4000

^(1)^ Abcam: Cambrigde, UK; Biorad: Munich, Germany; Cell Signaling Technology B.V.: Frankfurt, Germany; Exbio: Prague, Czech Republic; ^(2)^ HRP: horseradish peroxidase.

## Data Availability

The original data used to support the findings of this study are either included in the article or are available from the corresponding author upon request.

## Supplementary Materials

The following supporting information can be downloaded at: https://www.mdpi.com/article/10.3390/biom12050734/s1, Original images of Western blot analyses, of which cropped images are shown in Figure 1 and Figure 2, can be found in Figures S1 and S2, respectively.
