# Supplementary material for: Impairment of the Retinal Endothelial Cell Barrier Induced by Long-Term Treatment with VEGF-A165 No Longer Depends on the Growth Factor’s Presence
Source: Biomolecules. 2022 May 23;12(5):734. doi: 10.3390/biom12050734 (PMC9138398; doi:10.3390/biom12050734)

Supplementary Material:

Impairment of the retinal endothelial cell barrier induced by long-term treatment with VEGF-A_{165} is independent of the growth factor's action

Heidrun L. Deissler, Matus Rehak and Armin Wolf

<u>Original images of Western-blot analyses:</u>

Proteins (whole cell extracts or protein fractions) were separated by SDS-PAGE and transferred to a PVDF-Membrane which was then incubated with the appropriate primary and secondary antibodies, followed by detection of the chemiluminescence signals.

To determine presence of multiple antigens in parallel, the protein-bound membrane was cut in two pieces (see following pages) and exposed to the mentioned antibodies. Afterwards the membrane parts were incubated for 45 min in Restore Plus Western Blot Stripping Buffer (ThermoFisher Scientific, #46430) at room temperature to remove bound antibodies. After washing three times for 5 min with 0.1% Tween-20/PBSd and blocking, the membrane parts were exposed to another set of antibodies. This procedure was followed for a second or third time, if necessary.

Chemiluminescence signals were always directly scanned with the imaging system Fusion Pulse TS (Vilbert Lourmat), a black-and-white image of the membrane containing the prestained size marker proteins (peqGOLD Protein Marker V, VWR, avantor) was taken in parallel. The inverse image of the antibody-specific signal (black bands on grey background) was merged with the corresponding image of the membrane containing the prestained size marker with EvolutionCapt software (Version 17.01; Vilbert Lourmat). Labels were added to the merged image using the editing-function of the software.

Figure S1: Original images of Figure 1

1. Cellular extracts were prepared from iBREC exposed to 50 ng/ml VEGF-A_{165} for **1 d and subsequently to 1 mg/ml brolucizumab for additional 8 d.**
2. Proteins were separated under reducing conditions by SDS-PAGE and transfered to a PVDF-membrane. Protein standard: peqGOLD Protein Marker V (Avantor)
3. Membrane was cut into two pieces just above 70 kDa (see arrow). After blocking, the upper part was exposed to antibodies specific for VEcadherin (VECad) and the lower part to antibodies specific for claudin-1 and chemiluminescence signals were scanned.

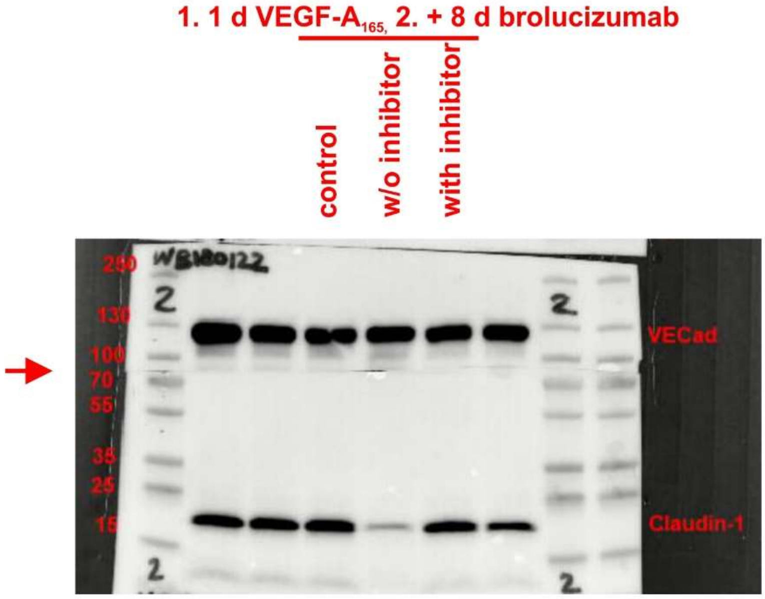

4. Both parts of the membrane were incubated with Restore Plus Western Blot Stripping Buffer for 45 min at RT, before they were blocked and incubated with antibodies against CD49e (upper part) or actin (lower part).

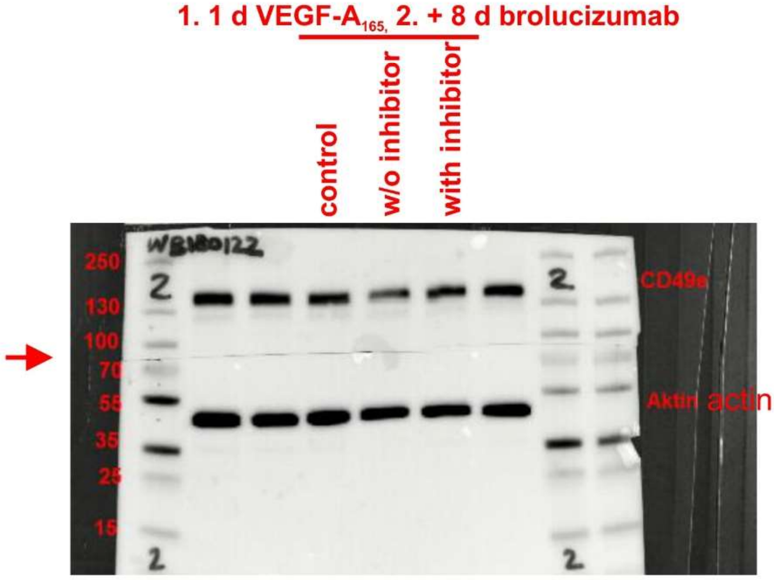

5. The lower part of the membrane was again incubated with Restore Plus Western Blot Stripping Buffer for 45 min at RT before it was blocked and incubated with antibodies against claudin-5.

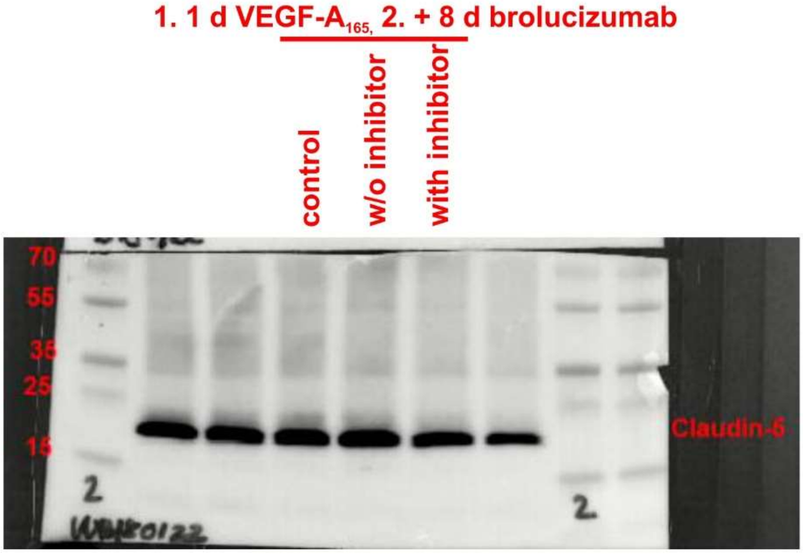

6. The lower part of the membrane was again incubated with Restore Plus Western Blot Stripping Buffer for 45 min at RT before it was blocked and incubated with antibodies against caveolin-1

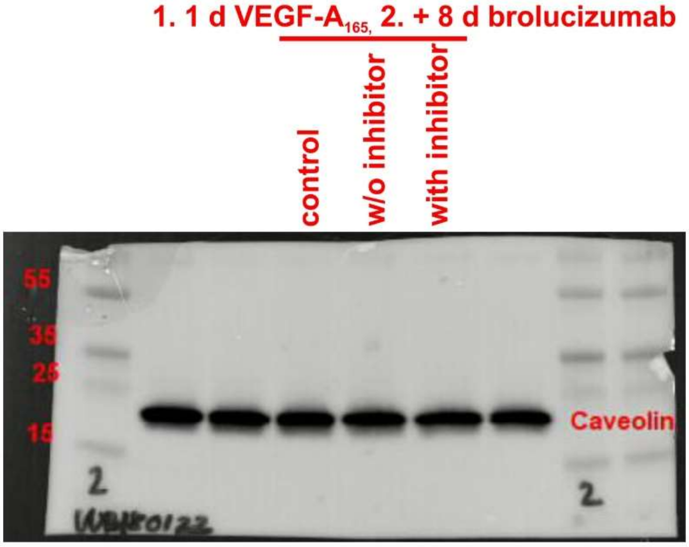

1. Cellular extracts were prepared from iBREC exposed to 50 ng/ml VEGF-A_{165} for **1 d and subsequently to 1 mg/ml brolucizumab for additional 8 d.**
2. Proteins were separated under *non-reducing* conditions by SDS-PAGE and transfered to a PVDF-membrane. Protein standard: peqGOLD Protein Marker V (Avantor)
3. Membrane was cut into two pieces just above 70 kDa (see arrow). After blocking, the upper part was exposed to antibodies specific for CD29 and the lower part to antibodies specific for VEGF-A and chemiluminescence signals were scanned.

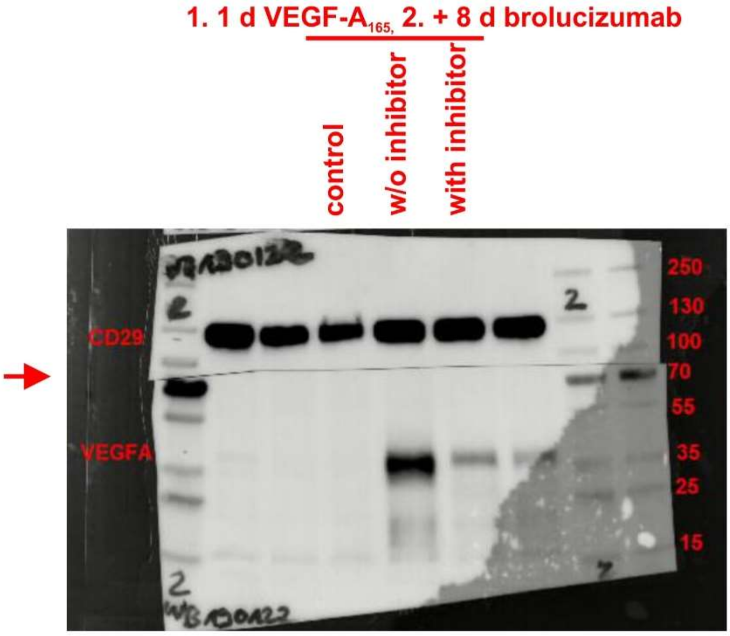

4. The lower part of the membrane was incubated with Restore Plus Western Blot Stripping Buffer for 45 min at RT before it was blocked and incubated with antibodies against actin.

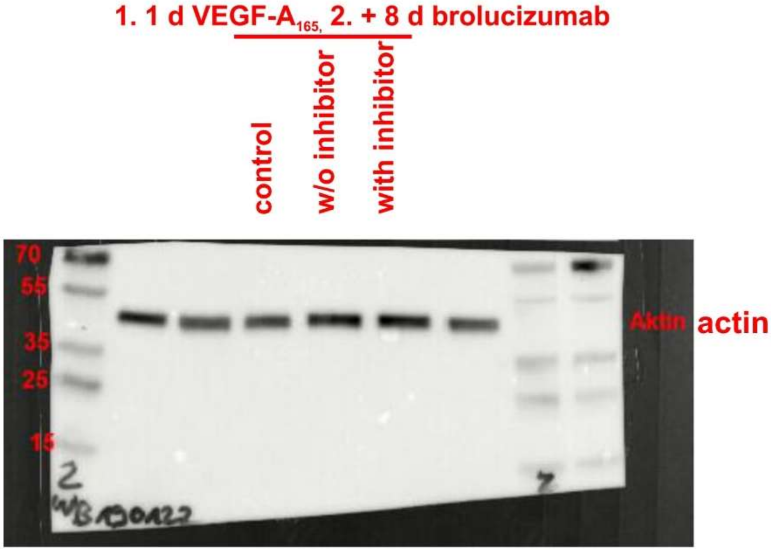

5. The lower part of the membrane was incubated with Restore Plus Western Blot Stripping Buffer for 45 min at RT, before it was blocked and incubated with antibodies against CD9. Signals specific for actin were not completely removed by stripping buffer and therefore additionally detected with secondary antibodies directed against mouse IgG used to measure the CD9-spedific signal.

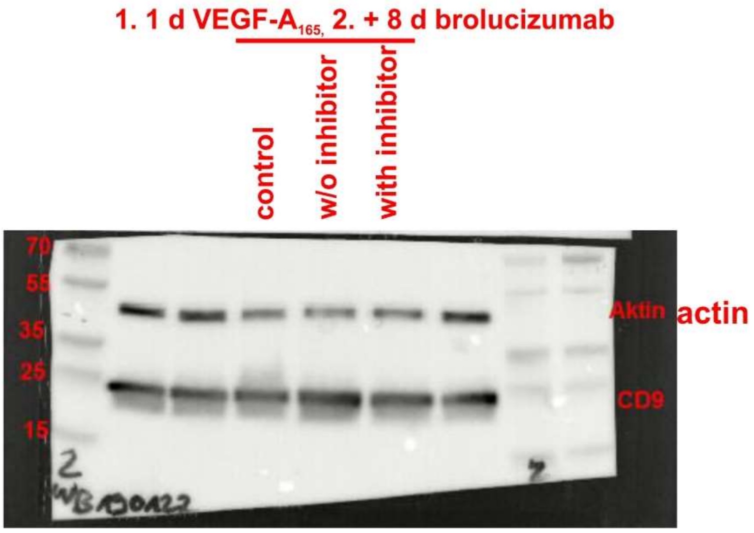

Figure S2 Original images of Figure 2

1. Cellular and nuclear extracts were prepared from iBREC exposed to 50 ng/ml VEGF-A_{165} for **1 d.**
2. Proteins were separated under reducing conditions by SDS-PAGE and transfered to a PVDF-membrane. Protein standard: peqGOLD Protein Marker V (Avantor)
3. Membrane was exposed to antibodies specific for β-catenin and chemiluminescence signals were scanned.

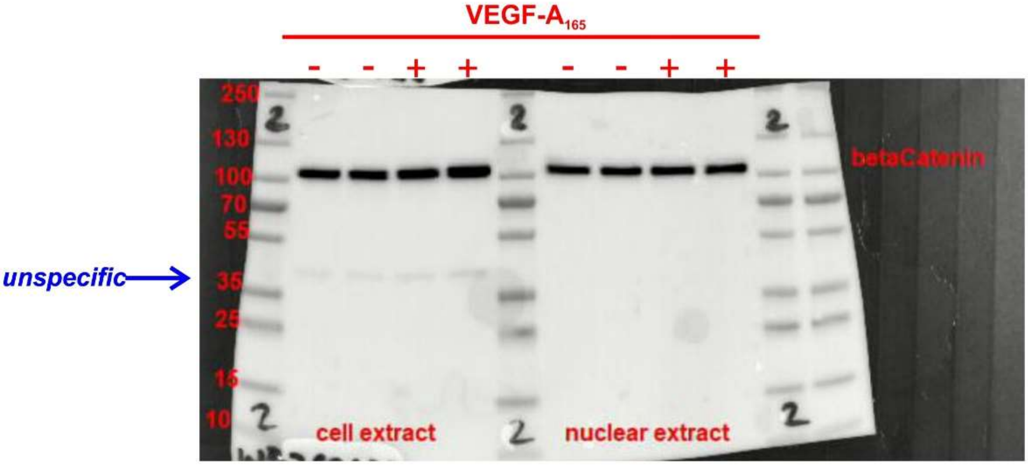

1. Cellular and nuclear extracts were prepared from iBREC exposed to 50 ng/ml VEGF-A_{165} for **2 d.**
2. Proteins were analyzed by Western-blotting as described above.

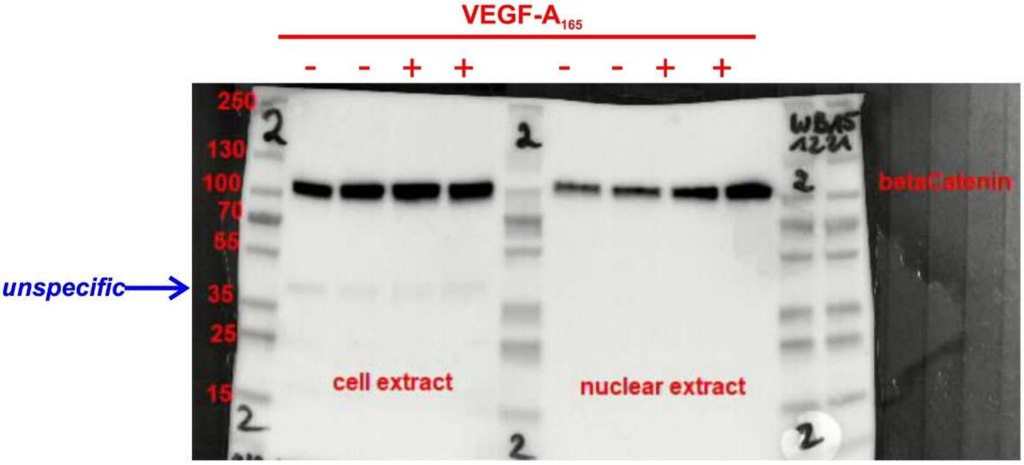

1. Cellular and nuclear extracts were prepared from iBREC exposed to 50 ng/ml VEGF-A_{165} for **3 d.**
2. Proteins were analyzed by Western-blotting as described above.

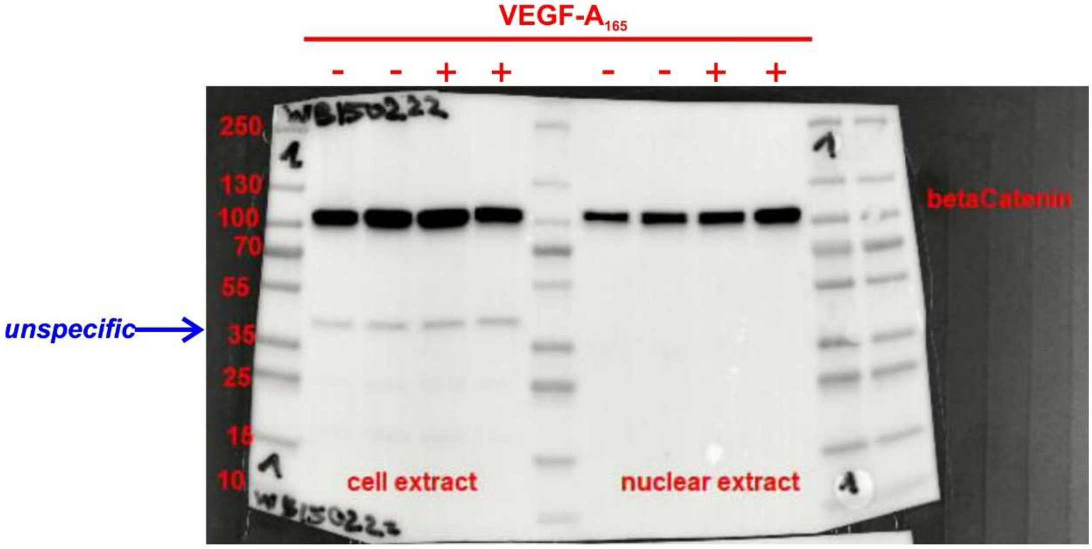

1. Cellular and nuclear extracts were prepared from iBREC exposed to 50 ng/ml VEGF-A_{165} for **6 d.**
2. Proteins were analyzed by Western-blotting as described above.

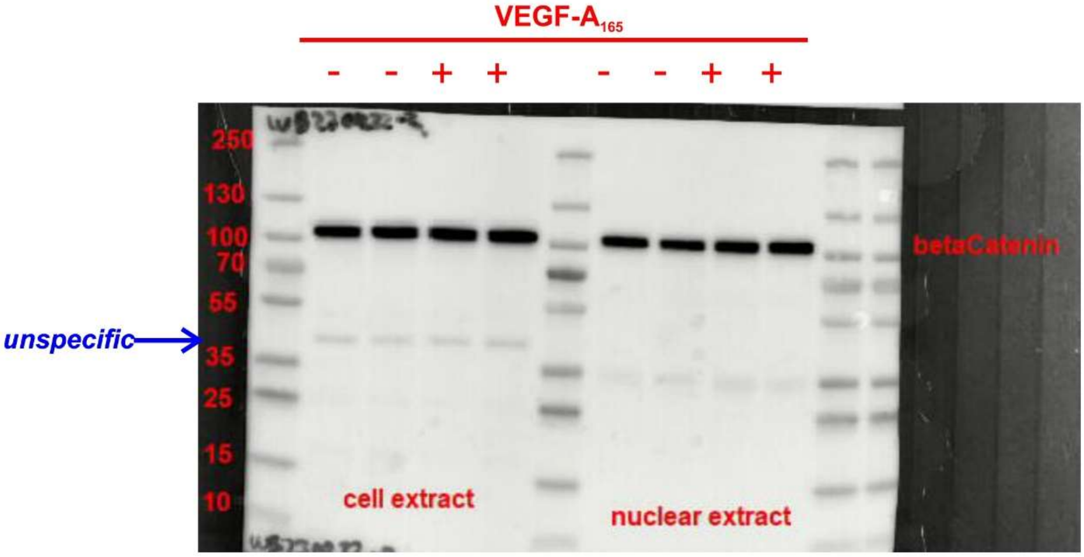

Supplement: Supplementary file 1 [file biomolecules-12-00734-s001.zip › biomolecules-1678864-supplementary.pdf]
